# Micro(mi) RNA-34a targets protein phosphatase (PP)1γ to regulate DNA damage tolerance

**DOI:** 10.1080/15384101.2015.1064202

**Published:** 2015-06-25

**Authors:** Yuko Takeda, Ashok R Venkitaraman

**Affiliations:** The Medical Research Council Cancer Unit; University of Cambridge; Cambridge, UK

**Keywords:** ATM, cancer, cancer therapy, DNA damage response, ionising radiation, miR-34a, miRNA, protein phosphatase, protein phosphatase 1γ, p53

## Abstract

The DNA damage response (DDR) triggers widespread changes in gene expression, mediated partly by alterations in micro(mi) RNA levels, whose nature and significance remain uncertain. Here, we report that miR-34a, which is upregulated during the DDR, modulates the expression of protein phosphatase 1γ (PP1γ) to regulate cellular tolerance to DNA damage. Multiple bio-informatic algorithms predict that miR-34a targets the *PP1CCC* gene encoding PP1γ protein. Ionising radiation (IR) decreases cellular expression of PP1γ in a dose-dependent manner. An miR-34a-mimic reduces cellular PP1γ protein. Conversely, an miR-34a inhibitor antagonizes IR-induced decreases in PP1γ protein expression. A wild-type (but not mutant) miR-34a seed match sequence from the 3′ untranslated region (UTR) of *PP1CCC* when transplanted to a luciferase reporter gene makes it responsive to an miR-34a-mimic. Thus, miR-34a upregulation during the DDR targets the 3′ UTR of *PP1CCC* to decrease PP1γ protein expression. PP1γ is known to antagonize DDR signaling via the ataxia-telangiectasia-mutated (ATM) kinase. Interestingly, we find that cells exposed to DNA damage become more sensitive – in an miR-34a-dependent manner – to a second challenge with damage. Increased sensitivity to the second challenge is marked by enhanced phosphorylation of ATM and p53, increased γH2AX formation, and increased cell death. Increased sensitivity can be partly recapitulated by a miR-34a-mimic, or antagonized by an miR-34a-inhibitor. Thus, our findings suggest a model in which damage-induced miR-34a induction reduces PP1γ expression and enhances ATM signaling to decrease tolerance to repeated genotoxic challenges. This mechanism has implications for tumor suppression and the response of cancers to therapeutic radiation.

## Introduction

Profound changes in gene expression occur when human cells are exposed to DNA damage, but their nature, mechanism and biological significance remain poorly understood. Most attention has focused on damage-activated transcription factors, including the tumor suppressor p53 or generic regulators of the transcriptional response to cellular stress including AP-1 or NFkB (eg.^1,2^) However, there is emerging evidence that damage-activated expression of miRNAs also plays a critical role in shaping changes in gene expression following the exposure of human cells to DNA damage. miRNAs elicit post-transcriptional gene regulation by directing the RNA-induced silencing complex (RISC) to a region on target mRNAs that is complementary to the so-called miRNA seed sequence spanning nucleotides (nt) 2–7 of the miRNA. The miR-34 family, comprising miR-34a, b and c, shares the same seed sequence, and is transcriptionally controlled by p53.^3^ In particular, miR-34a (which is the predominant species expressed in most tissues^4^) is a major substrate for p53-dependent regulation.^3,5,6^ Consistent with p53 function, miR-34 family members act as tumor suppressor miRNAs, by silencing the expression of several growth-promoting cellular oncogenes, including *MET, CDK6*, and *E2F* to induce G_1_ arrest, apoptosis or senescence in different cellular contexts.^4^ Indeed, miR-34 family members are de-regulated in several different tumor types.^5,7-10^ These considerations prompted us to investigate further the potential biological role of miR-34a because miR-34a expression is upregulated during the human DDR in a p53-dependent manner.^3,11-13^

We report in this work that the enzyme PP1γis targeted by miR-34a, silencing its expression after DNA damage. PP1γ is one of 3 known isoforms of protein phosphatase 1, an important group of Ser/Thr phosphatases believed to be responsible for the majority of protein dephosphorylation reactions in eukaryotic cells.^14,15^ The 3 isoforms appear to be functionally redundant at least in part, since they share ∼85% amino acid similarity,^16^ and genetic ablation of any single isoform does not cause lethality in murine models.^17^ However, the regulatory subunits of PP1 family members endow them with notable functional specificity. Thus, the regulatory subunit of PP1γ – the so-called Repo-man protein.^18,19-22^ – contributes to specific roles of the enzyme in several cellular functions, including DNA damage checkpoint activation during interphase,^18^ dephosphorylation of histone H3T3 during metaphase,^20^ or in chromatin remodeling and reformation of nuclear envelope at mitotic exit.^19^ Accordingly, we investigated the functional significance of reduced PP1γ expression by damage-induced miR-34a induction.

Interestingly, we find here that chromatin-associated PP1γ expression declines after DNA damage to reach its nadir ∼72h afterwards, and that at this time, cells become more sensitive to a second challenge. Increased sensitivity to the second round of damage manifests in evidence that damage-induced signaling via ATM is enhanced, resulting in enhanced phosphorylation of ATM and p53, as well as increased γH2AX formation. Increased sensitivity also manifests in enhanced cell death induced by the second DNA damage challenge. These phenotypes can be partly recapitulated by a miR-34a-mimic, or antagonized by an miR-34a-inhibitor. Our findings therefore suggest a model in which cellular tolerance to multiple rounds of DNA damage is modulated via miR-34a-dependent changes in PP1γ expression, leading to increased ATM signaling. Such a mechanism may ensure the elimination of damaged cells to suppress tumor formation after repeated exposure to genotoxic stress, and also has implications for the treatment of cancer with therapeutic radiation.

## Results

### PP1CCC mRNA encoding PP1γ is predicted to be an miR-34a target

To discern possible functions of miR-34a induction during the DDR, we first attempted to identify potential mRNA targets, using 3 different algorithms that account for various features of miRNAs and mRNAs into account, which commonly include thermodynamic stability of a miRNA::mRNA duplex, seed region complementarity, location of a seed region, target site accessibility, and sequence conservation among different species. For this approach, we used 3 different programs: miRanda,^23^ picTar4 and picTar5.^24^ In brief, miRanda scores miRNA: mRNA alignment based on sum of the match values using following base-pair values: A:U = 5, G:C = 5, G:U = 1, and all other base pairs (mismatches) = −3.^25^ picTar4 and picTar5 are 2 different versions of the same prediction program, which in addition to other features account for conservation between 4 (human, mouse, rat, and dog; picTar4) or 5 (human, mouse, rat, dog, and chicken; picTar5) species respectively.^26^ picTar4 assesses conservation among fewer species, but is biased toward identifying predictions of higher specificity in exchange for higher sensitivity, due to the inverse correlation between these factors.^42^

**Figure 1.**
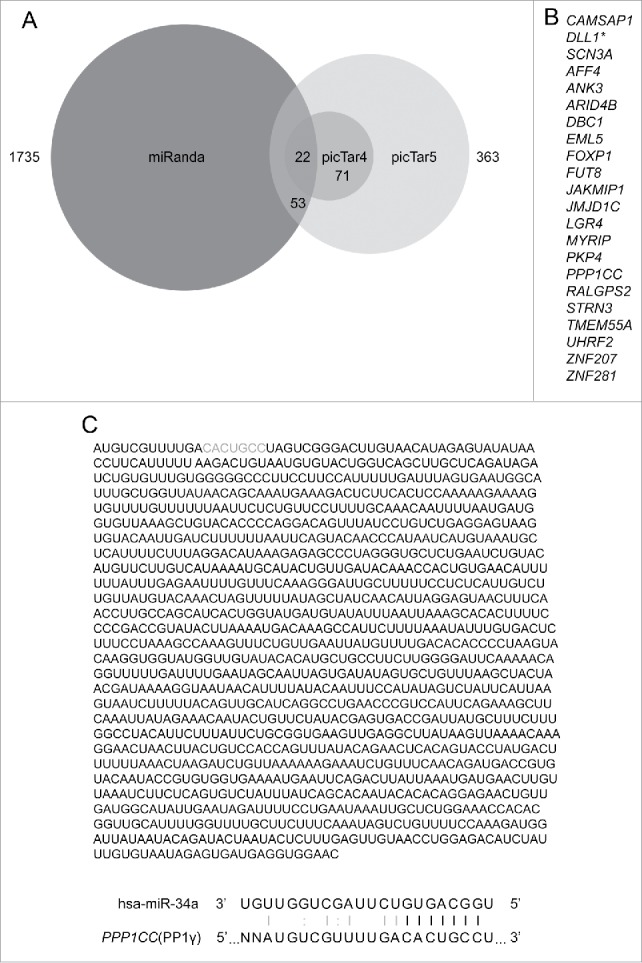
Bioinformatical prediction of miR-34a target genes. (**A**) Three-way Venn diagram illustrating the numbers of miR-34a targets predicted by miRanda, picTar4, and picTar5 programs. (**B**) List of miR-34a targets predicted by all 3 target prediction programs (*=target that has previously been confirmed as an miR-34a target). (**C**) Whole sequence of PP1γ 3′UTR and details of the seed match with miR-34a. Gray bases indicate the sequence that matches the miR-34a seed. Black lines between bases represent seed match. Gray line and colon represent base pair and wobble-base pair respectively.

A total of 22 overlapping targets identified by all 3 algorithms (**Fig. 1A and B**) were considered for further investigation. The list includes *DLL1*, a known target of miR-34a,^43^ illustrating the feasibility of the prediction approach. Eight previously unreognized predicted targets were confirmed to have miR-34a seed sequence(s) in their 3′UTR using the TargetScan program (**Table S1**). Of these, PP1γ, a protein encoded by *PPP1CC*, has been previously implicated in DNA damage checkpoint activation; it dephosphorylates Ser1981 of pATM throughout interphase, thereby antagonizing ATM-dependent signal transduction.^27^ The predicted miR-34a seed match sequence within PP1γ mRNA is positioned between bases 11–17 of its 3′ UTR, with an additional Watson-Crick base pairing and wobble base pairing outside the seed match sequence (**Fig. 1C and D**).

### PP1γ mRNA and protein downregulation accompany damage-induced miR-34a induction

**Figure 2.**
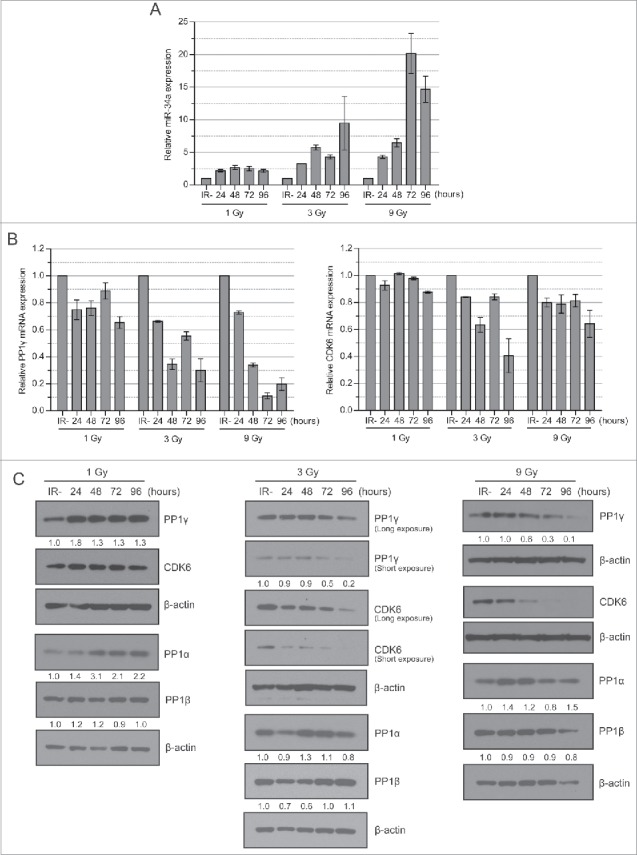
Dose-dependent miR-34a induction after IR is accompanied by down-regulation of PP1γ mRNA and protein. (**A**) Cal51 cells were irradiated with 1, 3 and 9 Gy and were harvested every 24 h up to 96 h. The 0 h sample not exposed to IR was deemed as a negative control. miR-34a expression was studied by qRT-PCR (n = 3; ±SEM). (**B**) PP1γ and CDK6 mRNA expression was studied by qRT-PCR (n = 3; ±SEM). (**C**) Western blot analysis. Numbers shown below PP1γ, PP1α and PP1β blots are densitometry values representing relative protein expression.

To address whether PP1γ mRNA and protein downregulation accompany damage-induced miR-34a induction, we studied their expression in CAL51 cells exposed to 1, 3 or 9 Gy of IR. RNA prepared from cell extracts harvested every 24 h from 0h – 96 h post-irradiation was subjected to quantitative reverse-transcription polymerase chain reaction (qRT-PCR) analysis for levels of miR-34a and PP1γ mRNA expression, while a known miR-34a target, CDK6,^28^ was studied as a positive control (**Fig. 2B and C**). miR-34a levels exhibited a dose-dependent increase, with maximal induction to 20.1 fold (SEM = ±3.1, n = 3) higher than basal levels by 72 h after exposure to 9 Gy IR (**Fig. 2A**). By comparison, 1 Gy irradiation induced an 2.7 (±0.3, n = 3) fold increase in miR-34a expression by 24 h after IR, while 3 Gy induced an approximately 9.5 (±4.1, n = 3) fold induction by 48 h after IR (**Fig. 2A**). Notably, PP1γ mRNA expression in the same samples was inversely correlated with miR-34a induction, reaching a nadir of ∼0.2 fold lower than basal expression by 72 h after exposure to 9 Gy IR (**Fig. 2B**). A lower dose of 1 Gy IR had little measurable effect on PP1γ mRNA expression, consistent with markedly lower induction of miR-34a at the same dose. CDK6 mRNA levels also decreased after IR, albeit less sharply than observed with PP1γ mRNA (**Fig. 2B**).

**Figure 3.**
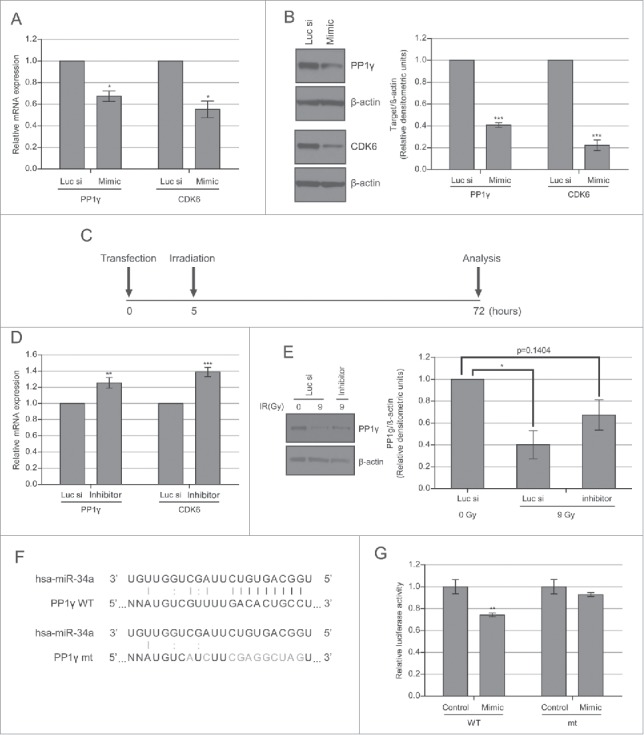
PP1γ is a target of miR-34a. (**A**) Cal51 cells were transfected with 50 nM miR-34a mimic or Luc siRNA and harvested 72 h post-transfection for analysis. qRT-PCR of PP1γ and CDK6 was performed (one-way ANOVA with Dunett's post-test; *P < 0.05) (n = 4; ±SEM). (**B**) Western blot analysis. The densitometry graph was generated from 4 independent experiments. (2-way ANOVA with Bonferroni's post-test; ***P < 0.001) (n = 4; ±SEM). (**C**) Experimental scheme. Cal51 cells were irradiated with 9 Gy, 5 h after transfection with miR-34a inhibitor or Luc siRNA. The cells were analyzed 72 h post-transfection. (**D**) qRT-PCR of PP1γ and CDK6 mRNA expression. The plotted values show the mean ±SEM (one-way ANOVA with Dunett's post-test; **P < 0.01, ***P**≤** 0.001, respectively) (n = 4). (**E**) Western blot analysis of the miR-34a inhibitor experiment. The densitometry graph shows the mean ±SEM of 3 independent experiments. The values of the samples were first normalized to β-actin, and then subsequently normalized to 0 Gy Luc siRNA transfected samples (2-way ANOVA with Bonferroni's post-test; *P < 0.05) (n = 3). (**F**) Schematic showing psiCHECK2-PP1γ_3′UTR constructs with either the wild-type (WT) or mutant (mt) forms of the predicted miR-34a seed match sequence in the PP1γ 3′UTR. Black lines between bases represent seed match. Gray line and colon represent base pair and wobble-base pair respectively. Bases in gray represent mutations introduced to disrupt matches between miR-34a and PP1γ. (**G**) Cal51 cells were co-transfected with psiCHECK2-PP1γ_3′UTR WT or mt and negative control miRNA or miR-34a mimic. Fluorescence was measured 72 h post-transfection. Reporter Renilla fluorescence values were normalized to Firefly luciferase fluorescence encoded in the same plasmid. Plots show the mean ±SEM (2 tailed t-test with Bonferroni's post-test; **P ≤0.01) (n = 4).

Next, we used Western blotting to determine PP1γ or CDK6 protein expression in extracts prepared from the same samples, using antibodies that specifically recognize each of the proteins (**Fig. S1A**). PP1γ protein expression was little affected after 1 Gy IR, but was decreased with 3 Gy and 9 Gy in a dose-dependent manner from the 72 h timepoint (**Fig. 2C**). Reduced CDK6 protein expression occurred somewhat earlier (**Fig. 2C**), and did not closely mirror reductions in mRNA levels (**Fig. 2B**), raising the possibility that miR-34a may act at a later stage during gene expression to affect CDK6 mRNA translation, as reported for other miR species.^29^ In contrast, PP1α and β protein levels showed little variation after IR (**Fig. 2C**), suggesting that this response is specific to PP1γ.

Consistent with previous reports,^3,11-13^ we find that damage-induced miR-34a induction depends on p53 (**Fig. S2**). In Cal51 cells, depletion of p53 using short interfering (si)RNAs decreases by >3-fold the induction of miR-34a expression at 72h after exposure to 3 Gy IR. Moreover, it has been reported that neuronal differentiation and synaptic outgrowth are regulated via the p73-mediated regulation of miR-34a in neuronal cells,^30,31^ raising the possibility that p73 or p63 may also participate in miR-34a regulation after DNA damage. However, we have been unable to directly test this possibility owing to technical difficulties in achieving efficient and selective depletion of different p63 and p73 isoforms in our experimental setting.

### An miR-34a mimic reduces PP1γ protein expression

We transfected a mimic of miR-34a into CAL51 cells to determine effects on PP1γ expression. Analysis of the transfected cells by qRT-PCR and western blot revealed that the overexpression of miR-34a mimic suppresses PP1γ expression at both mRNA and protein level (**Fig. 3A, B, and Fig. S1B, C**). Decreased PP1γ mRNA expression reaches its nadir about 48 h after miR-34a mimic transfection, and remains at this level (**Fig. S3B** **and** **C**). However, the magnitude of protein downregulation (∼60%) was somewhat higher than that of mRNA (∼35%), raising the possibility that the miR-34a-mimic may in part regulate PP1γ expression at the translational level. These findings confirm that PP1γ mRNA and protein are indeed reduced by an miR-34a mimic, consistent with the notion that their expression is targeted by this miR species.

### An miR-34a inhibitor counteracts the damage-induced reduction in PP1γ protein expression

To confirm that miR-34a induction after DNA damage is responsible for the observed decrease in PP1γ expression, we asked if an inhibitor of miR-34a could counteract this effect. We transfected CAL51 cells with miR-34a inhibitor before exposure to 9 Gy IR, the dose at which the highest downregulation of PP1γ is observed at 72–96 h after exposure (**Fig. 2B**). Extracts prepared from miR-34a inhibitor-treated cells 72 h after IR exposure were subjected to qRT-PCR and Western blot analysis (**Fig. 3C**). Damage-induced decreases in PP1γ mRNA and protein expression were counteracted in part by the miR-34a inhibitor (**Fig. 3D and E**). For PP1γ protein, treatment with the miR-34a inhibitor caused an approximately 2-fold increase in expression, to about half the level of basal expression.

**Figure 4.**
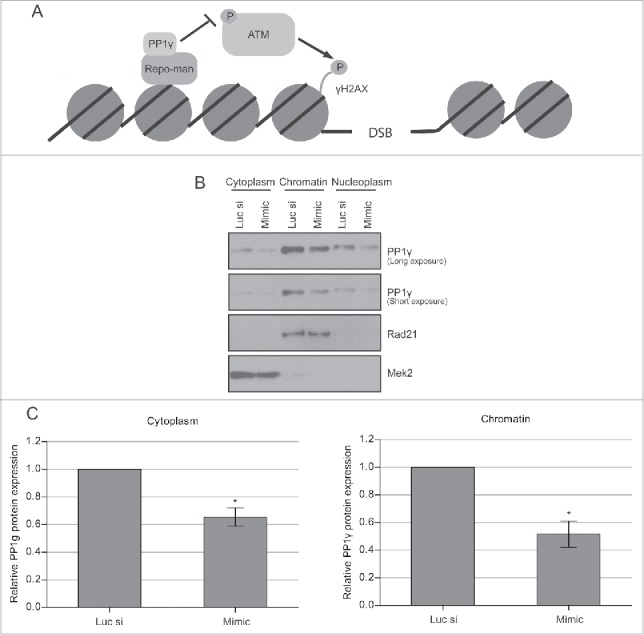
Decreased PP1γ protein expression in different cellular fractions after IR. (**A**) Schematic depiction of the PP1γ. ATM interaction. PP1γ bound to its regulatory subunit Repo-man is proposed to inhibit ATM phosphorylation and activation via colocation on chromatin. (**B**) Cytoplasmic, nucleoplasmic, and chromatin protein fractions from Cal51 cells were prepared 72 h after transfection with either Luc siRNA or miR-34a mimic, and the PP1γ protein level was determined by western blotting. (**C**) The densitometry graph shows relative PP1γ protein expression in each lane normalized to the corresponding loading control. Plots show the mean ±SEM (2-way ANOVA with Bonferroni's post-test, *P < 0.05) (n = 3).

### Transplantation of wild-type and mutant miR-34a seed match sequences to a luciferase reporter gene

Our results thus far show that DNA damage-induced upregulation of miR-34a is accompanied by decreased PP1γ expression (**Fig. 2B and C**), that the overexpression of an miR-34a mimic suppresses PP1γexpression (**Fig. 3A and B**), and that an miR-34a inhibitor partially counteracts damage-induced reductions in PP1γ mRNA and protein expression (**Fig. 4C–E**). Together, these results provide strong evidence that damage-induced miR-34a upregulation silences PP1γ expression. To further validate this conclusion, we tested whether the miR-34a seed match sequence from the 3′ UTR of PP1γ mRNA could make a heterologous luciferase reporter gene become miR-34a responsive. To this end, we inserted either wild-type (WT) or mutant forms of the miR-34a seed match sequence the 3′UTR of PP1γ downstream of the *Renilla* luciferase gene. The seed match sequence and some complementary bases outside the seed match sequence were mutated to generate a mutant plasmid (**Fig. 3F**). The luminescence of miR-34a mimic or negative control miRNA transfected cells co-transfected with the predicted seed match sequence WT or mutant plasmid was then measured. Approximately 30% lower *Renilla* luciferase activity was detected in the samples co-transfected with WT plasmid and miR-34a mimic, compared to the samples co-transfected with WT plasmid and negative control miRNA (**Fig. 3G**). By contrast, there was no significant difference in *Renilla* luciferase activity between the samples co-transfected with mutant plasmid and miR-34a mimic or negative control miRNA, confirming that miR-34a directly targets PP1γ through PP1γ 3′UTR seed match sequence (**Fig. 3G**).

**Figure 5.**
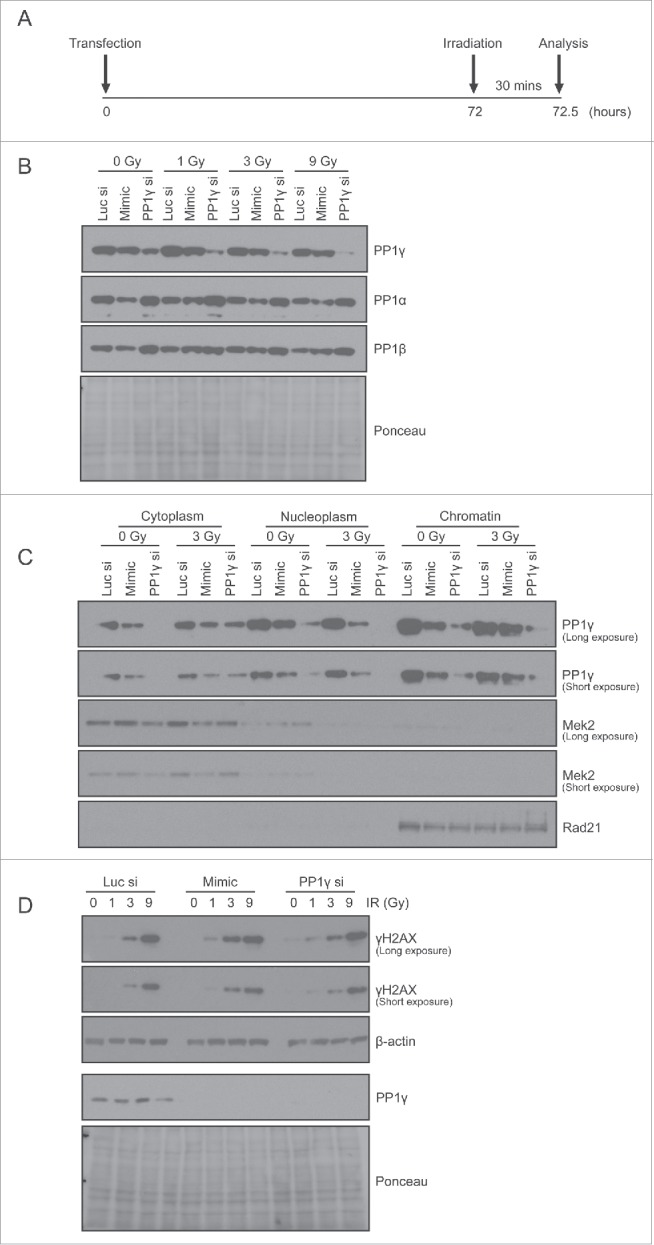
PP1γ protein downregulation by miR-34a mimic sensitizes cells to DNA damage. (**A**) Experimental scheme. At 72 h after transfection with Luc siRNA, miR-34a mimic or PP1γ siRNA, Cal51 cells were irradiated with 1, 3 or 9 Gy, and harvested 30 mins post-IR for analysis. (**B–D**) Analysis by Western blot after IR exposure of PP1 isoform expression, PP1g in different cellular fractions, and γH2AX expression.

### Damage-induced reduction in PP1γ via miR-34a enhances ATM signaling

These observations led us to consider the potential biological significance of miR-34a-induced reductions in PP1 expression after DNA damage. PP1γ is directed onto chromatin upon anaphase onset by its regulatory subunit Repo-man, forming a PP1γ-Repo-man holoenzyme.^27^ The holoenzyme dephosphorylates ATM throughout interphase (**Fig. 4A**), suppressing downstream signal transduction,^27^ and is dispersed into the cytoplasm at the beginning of prophase.^22,44^ This function appears to be specific to PP1γ, because it is the only one of the 3 PP1 isoforms that is localized to chromatin via Repo-man.^27^

The localization of PP1γ protein to chromatin, nucleus and cytoplasm led us to ascertain whether miR-34a-dependent reductions in expression affected both compartments. We therefore prepared extracts from the corresponding compartments in CAL51 cells exposed to miR-34a-mimic, or control RNA, for 72 h before protein gel blotting with an antibody against PP1γ. PP1γ is expressed in all of the subcellular fractions studied (**Fig. 4B and C**). The highest expression was observed in the chromatin fraction (∼60% of the total cellular PP1γ) while the cytoplasmic fraction had the lowest expression (∼10% of total protein). Exposure to the miR-34a mimic suppressed PP1γ by about 2-fold on chromatin and by about 1.6-fold in the cytoplasm, relative to their corresponding negative control siRNA transfected samples, normalized to a designated subcellular marker (**Fig. 4B and C**). These results suggest that miR-34a reduces PP1γ protein expression in all subcellular compartments.

We find that miR-34a induction after IR rises 24–48 h after exposure to reach its peak by about 72–96 h (**Fig. 2A**). Thus, miR-34a induction is *not* an immediate or early response to DNA damage, and so its effect on PP1γ expression is not expected to affect DNA damage signaling via ATM after a first exposure, because ATM-mediated events initiate within minutes.^32^ This led us to ask whether the ATM response might be impaired 72 h after the exposure of cells to miR-34a mimic, when PP1γ expression reaches its nadir.

We first chose to investigate the effect of miR-34a-mimic on the formation of γH2AX, a phosphorylated form of the variant histone H2AX, which is directly modified by ATM within minutes of DNA damage, and serves as a well-established marker for downstream signaling via the ATM pathway.^33^ Damage was administered with IR, which induces DNA breaks that predominantly activate ATM rather than other related enzymes like ATM and Rad53-related kinase, ATR.^34^ Accordingly, CAL51 cells exposed to miR-34a-mimic were incubated for 72 h to allow PP1γ suppression, before exposure to 1, 3 or 9 Gy IR, and assessment of γH2AX formation 30 min afterwards (**Fig. 5A**). Cell lysates were subjected to Western blot analysis. PP1γ protein expression is downregulated by exposure to the miR-34a mimic (as well as with PP1γ siRNA used as a positive control) (**Fig. 5B**). Levels of PP1α or PP1β were not greatly affected. Moreover, fractionation of cell extracts exposed to 3 Gy IR showed a downregulation of chromatin-localized PP1γ, as well as PP1γ in nucleoplasmic and cytoplasmic compartments, in both miR-34a mimic and PP1γ siRNA transfected samples (**Fig. 5C**). There was no significant effect of miR-34a mimic or PP1γ siRNA on cell cycle distribution analyzed by flow cytometry (data not shown). Notably, both miR-34a mimic or PP1γ siRNA transfected samples exhibited a higher γH2AX level than controls exposed to 1 or 3 Gy (**Fig. 5D**), indicating that ATM signaling had indeed been enhanced via PP1γ suppression.

### Damage-induced miR-34a induction enhances ATM signaling to a second challenge via PP1γ suppression

**Figure 6.**
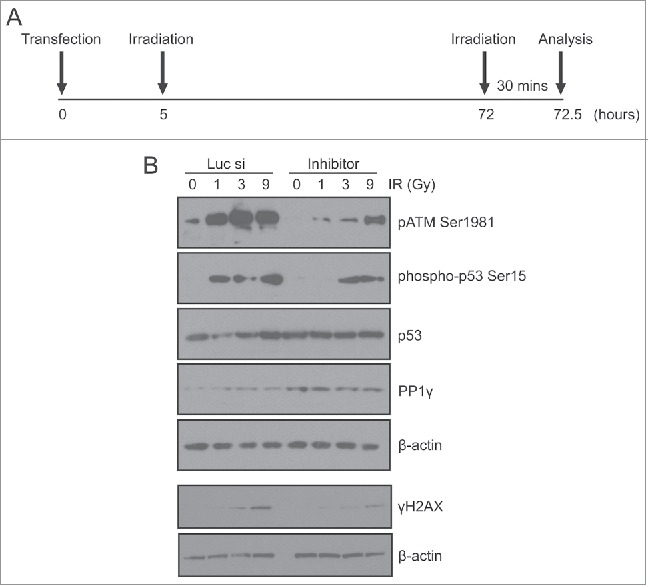
An miR-34a inhibitor counteracts PP1γ protein downregulation and increased ATM signaling after a second challenge with DNA damage. (**A**) Experimental scheme. Cal51 cells transfected with Luc siRNA or miR-34a inhibitor were irradiated with 3 Gy at 5 h post-transfection. A second challenge with irradiation (1, 3 or 9 Gy) was given at 72 h post-transfection. The cells were harvested for analysis 30 min after the second IR challenge. (**B**) Analysis by Western blot of markers of ATM signaling (pATM Ser1981, phospho-p53 Ser15, γH2AX) and PP1γ expression after a second challenge with IR in cells exposed to Luc siRNA or miR-34a inhibitor.

These observations led us to ask whether damage-induced decreases in PP1γ expression mediated by miR-34a might affect ATM signaling in cells exposed to a second challenge with DNA damage, administered 72 h after the first exposure, to coincide with the peak effects of miR-34a induction on PP1γ expression. To address this issue, we transfected CAL51 cells with miR-34a inhibitor or negative control siRNA, then irradiated the cells with 3 Gy approximately 5 h post-transfection to induce miR-34a. After incubating the cells for 72 h post-3 Gy IR, they were irradiated again with 1, 3, or 9 Gy IR, with cells not exposed to IR (IR-) deemed as the negative control (**Fig. 6A**). The cells were incubated for 30 min after the second round of irradiation the preparation of cell extracts for Western blot analyses. There was no significant difference in the cell cycle profile between different samples exposed to 3 Gy IR (data not shown). Importantly, samples transfected with miR-34a inhibitor exhibited decreased ATM signaling after 1 or 3 Gy IR, marked by decreases in γH2AX formation, and the appearance of the ATM pSer1981 activation marker,^35^ when compared to samples transfected with negative control siRNA (**Fig. 6B**). Unlike γH2AX, the difference in pATM level was evident even in the unirradiated negative control samples, suggesting that some pATM activity might be carried over from the first round of irradiation. We also studied the phosphorylation of another ATM target, p-p53 Ser15. Although total p53 expression is not altered after a second challenge with IR, the levels of p-p53 Ser15 exhibited a dose-dependent increase in samples transfected with negative control siRNA transfected samples (**Fig. 6B**). However, p-p53 Ser 15 expression was decreased in samples transfected with miR-34a, again illustrating that ATM signaling is enhanced by damage-induced miR-34a, a delayed response to DNA damage that can be counteracted by the miR-34a inhibitor (**Fig. 6B**).

### Damage-induced miR-34a induction enhances cell death after a second challenge

**Figure 7.**
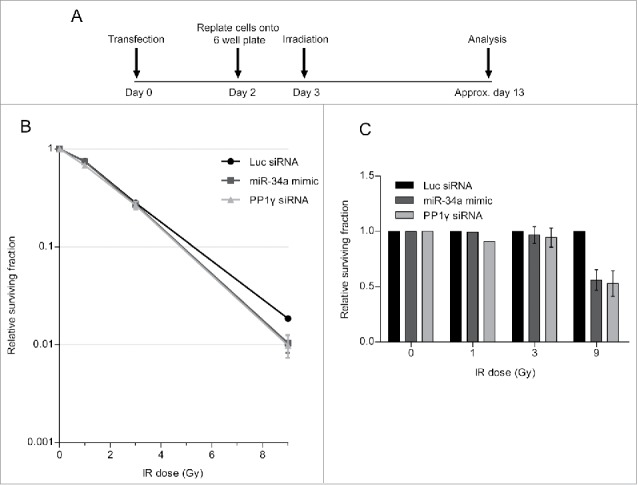
PP1 depletion using siRNA or an miR-34a mimic decreases clonogenic cell survival after DNA damage. (**A**) Experimental scheme. Cal51 cells were transfected with Luc siRNA, miR-34a mimic or PP1γ siRNA. Cells were replated into 6-well plates at 48 h post-transfection. At 72 h post-transfection, the plates were irradiated (1, 3, or 9 Gy), incubated for further ˜10 days and subjected to crystal violet staining once they reached their optimal density. The colonies were then counted and analyzed. (**B**) Survival curves from a representative experiment (±SD). (**C**) Survival fraction values of each experimental sample were normalized to those from the corresponding Luc siRNA transfected samples exposed to the same IR dose (2-way ANOVA with Bonferroni's post-test; ***P < 0.001) (mean ± SEM; n = 2).

**Figure 8.**
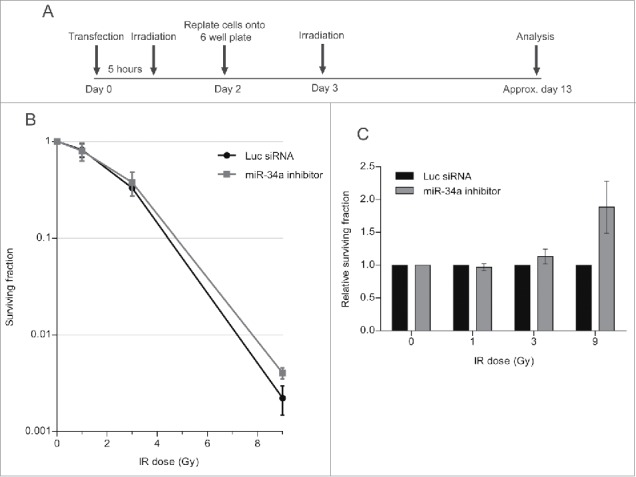
An miR-34a inhibitor counteracts decreased clonogenic cell survival after a second challenge with DNA damage. (**A**) Experimental scheme. Cal51 cells were transfected with Luc siRNA or miR-34a inhibitor and were irradiated with 3 Gy 5 h post-transfection. Cells were replated into 6-well plates at 48 h post-transfection. At 72 h post-transfection, the plates were irradiated for a second time (with 1, 3, or 9 Gy), and then incubated for further ˜10 days and subjected to crystal violet staining once they reached optimal density. The colonies were then counted and analyzed. (**B**) Survival curves of a representative experiment (±SD). (**C**) Survival fraction values of miR-34a inhibitor transfected samples were normalized to those from the corresponding Luc siRNA transfected samples exposed to the same IR dose (2-way ANOVA with Bonferroni's post-test; ***P < 0.001) (mean ± SEM; n = 3).

Next, we carried out a colony formation assay (CFA), which identifies the post-irradiation survival of cells which have retained the capacity to give rise to a progeny of at least 50 cells.^36^ The experiment was carried out as before, but cells were replated after the second challenge with IR at a low density in 6-well plates to allow the formation of colonies. The plates were incubated until the colonies were visible but not confluent, before staining with crystal violet dye for quantification (**Fig. 7A**). Results are plotted in the graph, which shows the surviving fraction as a ratio of the colony number in experimental samples normalized to unirradiated controls. Our findings (**Fig. 7B and C**) shows that cell survival after the second challenge with IR is reduced when cells are transfected with either the miR-34a-mimic, or with PP1γ siRNA, both of which we have shown to reduce PP1γ protein expression. This effect is statistically significant when cells are exposed to 9 Gy IR in the second challenge, but not at lower doses. Moreover, a similar experiment in which cells have been transfected with miR-34a inhibitor (**Fig. 8A**), which we have shown to counteract radiation-induced reductions in PP1γ protein expression, increases cell survival after the second IR challenge (**Fig. 8B and C**). Again, the difference is statistically significant at 9 Gy but not at lower doses. These findings suggest that damage-induced miR-34a induction enhances cell death after a second challenge with IR, consistent with its observed effects on ATM signaling.

## Discussion

**Figure 9.**
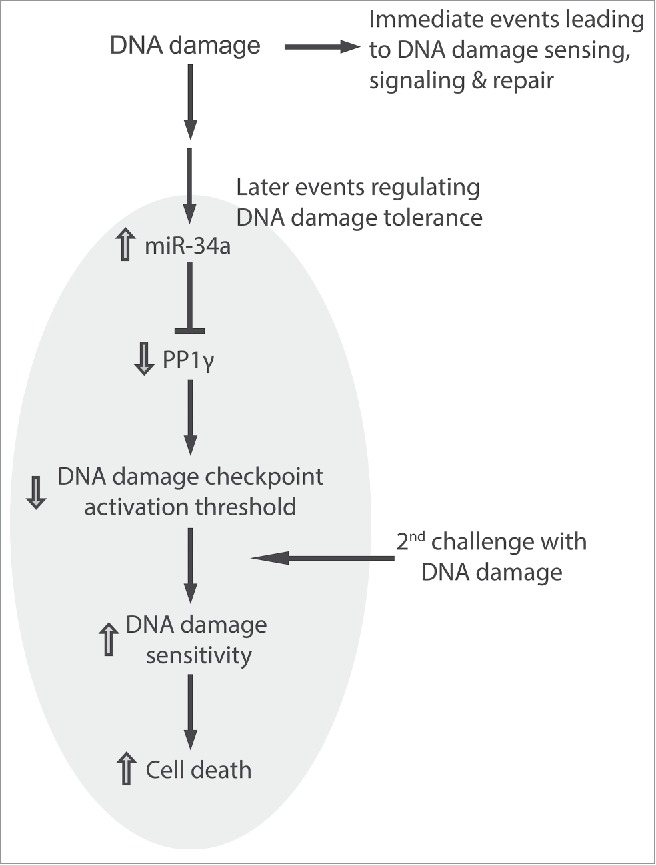
miR-34a regulates DNA damage tolerance by targeting PP1γ. The figure illustrates the model suggested by our findings wherein the induction of miR-34a expression by DNA damage acts via PP1γ to decrease the threshold for damage signaling via ATM. Immediate events at the site of damage lead to DNA damage repair. The induction of miR-34a and consequent downregulation of PP1γ, peaking some 72 h after exposure to damage, is a late event that alters cellular tolerance to increase sensitivity to a second challenge with DNA damage.

Our findings suggest a novel biological role for miR-34a induction following DNA damage. We propose (**Fig. 9**) that miR-34a induction, which reaches a peak some 72 h after induction, increases the sensitivity of cells to a second challenge with DNA damage, by decreasing the expression of PP1γ protein and thereby enhancing signaling via the damage-responsive ATM kinase. Our proposal has several noteworthy implications.

First, it reveals a previously unrecognized mechanism by which the changes in miRNA expression that have recently been shown to occur after DNA damage can influence the biological response to these lesions. Indeed, expression of the miR-99 family has been previously shown to radiosensitize cells through the downregulation of SNF2H, a facilitator of DNA double-strand break repair,^37^ suggesting that damage-induced miRNAs may orchestrate a complex network of alterations in the expression of proteins that participate in the DNA damage response. Cell-type specificity in the patterns of damage-induced miRNA expression is noteworthy in this context^13^; while miR-99 expression is apparently restricted in its tissue distribution, miR-34a is widely expressed in a p53-dependent manner.^38^

Second, our results suggest a biological significance for the kinetics of damage-induced miRNA expression. Whereas immediate events that lead to the sensing, signaling and repair of DNA damage begin within minutes,^32^ damage-induced changes in miRNA expression typically reach their peak many hours (24–72 h) after these immediate events. We provide several lines of evidence that miR-34a-induced changes in PP1γ protein expression some 72 h after damage can sensitize cells to a second challenge with damage, via the enhancement of ATM signaling. We speculate that such a response may “pre-arm” cells exposed to sub-lethal amounts of damage to subsequent, repeated challenges; “pre-arming” may be particularly important in metazoan organisms to preserve fitness through the elimination of damaged cells that might trigger carcinogenesis following repeated exposure to genotoxic stimuli.

Conversely, we speculate that miR-34a-induced “pre-arming” may render cancer cells more sensitive to fractionated therapeutic radiation (which is administered in multiple small doses over a period of time). Fractionated radiotherapy is more efficacious than single-dose therapy, but the mechanism underlying this remains unclear, although several factors have been implicated.^37^ If so, our work suggests that the integrity of the p53 pathway, which is reported to be necessary for damage-induced miR-34a induction,^39^ may be essential. Thus, the new mechanism that we report in this work has clinical implications for cancer radiotherapy that warrant further investigation. *In vivo* investigation of this notion using siRNA is warranted, but technically challenging owing to the difficulties in prolonged suppression of target expression with this approach,^40^ and so alternative approaches may be required.

## Materials & Methods

### Cell lines

CAL51 (human basal B subtype breast cancer cell line) was cultured in Dulbecco's Modified Eagle's Medium with GlutaMAX™-1, 4500 mg/L D-glucose, sodium pyruvate and pyridoxine (Life Technologies), supplemented with 10% fetal bovine serum (Life Technologies) and 100 units/ml penicillin-streptomycin (Life Technologies). The cells were maintained in a humidified incubator at 37°C under 5% CO_2_.

### X-ray irradiation

X-ray irradiation was performed by exposing cultured cells to irradiation using Faxitron RX-650 (Faxitron Bioptics; irradiation dose rates of 0.492, 0.358, and 0.211 Gy/min) at 120 kVp at 5 mA at room temperature.

### Transient transfection

Cells were transfected with plasmid and siRNA or miRNA mimic/inhibitor using Lipofectamine 2000™ (Life Technologies) and Dharmafect I (Life Technologies) respectively, following manufacturer's instructions. The details of siRNA and miRNA mimic/inhibitor are provided in supplementary table.

### Western blotting

The harvested cells were washed in PBS and lysed in NP-40 buffer (50 mM HEPES pH 7.4, 100 mM NaCl, 0.5% NP-40, 10 mM EDTA, 20 mM β-Glycerophosphate, supplemented with 1 mM DTT, 1 mM PMSF and 1 x protease inhibitor (Amersham)). The cells were incubated on ice for 15 min. Lower concentration of EDTA (1 mM) was used instead of 10 mM EDTA when Benzonase Nuclease (Millipore) was added, as high concentration of EDTA inhibits its enzymatic activity. Benzonase Nuclease was supplemented at a concentration of 25 U/ml, and the cells were incubated on ice for 30 min to facilitate the reaction. The samples were centrifuged at 12,000 g for 15 min at 4°C. The supernatants were retained, placed on ice and subjected to protein quantification using Bicinchoninic Assay (BCA) (Sigma). An equal amount of protein was subjected to analysis. 4 x lithium dodecyl sulfate (LDS) loading buffer (Life Technologies) was diluted to 1 x and supplemented with 100 mM DTT. The buffer was mixed with the whole cell lysate and was denatured at 70°C for 10 min. Alternatively, the cells were sonicated in NP-40 buffer. Samples were resolved by pre-cast gels (Life Technologies). Protein was transferred to an activated Immobilon-P membrane (Millipore) or nitrocellulose membrane (Sigma), either wet or semi-dry. The membrane was blocked either with 5% non-fat milk or 3% BSA in TBS-Tween on a rocker for 30–60 min at room temperature. The membrane was then incubated with primary antibody diluted in either 5% milk or 3% BSA in TBS-Tween. The membrane was incubated for overnight at 4°C or 4 h at room temperature. After primary antibody incubation, the membrane was placed on an orbital shaker and washed in TBS-Tween for 3 × 5 min. The membrane was incubated with secondary antibody diluted at a ratio of 1:10,000 in either 5% milk or 3% BSA in TBS-Tween on an orbital shaker for 1 h at room temperature. Then, the membrane was again washed in TBS-Tween for 3 × 5 min. The protein of interest was detected by applying ECL or ECL plus (GE healthcare) following manufacturer's instructions. The membrane was wrapped with Saran and exposed to CL-XPosure Film (Thermo Scientific) for an appropriate amount of time to detect the protein of interest.

β-Actin was deemed as a loading control for whole cell western blot; Mek2 and Rad21 were deemed as a loading control for cytoplasmic and chromatin fraction protein gel blot respectively, unless otherwise stated.

### Cellular fractionation assay

Cellular fractionation assay was performed as previously described.^41^ Briefly, the harvested cells were washed in PBS. The cell pellet was resuspended in 1 ml of buffer A (10 mM HEPES pH 7.9, 10 mM KCl, 1.5 mM MgCl_2_, 0.34 M sucrose, 10% glycerol, 1 mM DTT, 1 mM PMSF, 1 x protease inhibitors, 5 mM NaV, 10 mM NaF and 1 uM Okadaic acid, diluted in dH_2_O) per 4 × 10^7^ cells, and 0.05% Triton X-100 was added. The sample was aspirated and dispensed gently to mix. Nuclei were collected by centrifuging at 1300 g for 4 min at 4°C. Supernatant (cytoplasmic fraction), was transferred to a fresh tube and clarified by centrifuging at 20,000 g for 15 min at 4°C. The nuclear pellet was washed in half the volume of buffer A used for the initial resuspension to remove cell debris and insoluble aggregates. The washed nuclear pellet was lysed in buffer B (3 mM EDTA, 0.2 mM EGTA, 1 mM DTT, 1 mM PMSF, 1 x protease inhibitor, 5 mM NaV, 10 mM NaF and 1 uM Okadaic acid, diluted in H_2_O) (0.8 volume of buffer A used for the initial resuspension) by aspirating and dispensing gently. It was then centrifuged at 1700 g for 4 min at 4°C to pellet chromatin, and its supernatant (nuclear fraction), was transferred to a fresh tube. Chromatin pellet was resuspended in half the volume of buffer A used for the initial resuspension, adjusted with 1 mM CaCl_2_ and 0.2 U micrococcal nuclease. It was incubated at 37°C for 5–10 min, EGTA was added to 1 mM to quench the reaction, and was centrifuged at 20,000 g for 10 min at 4°C to collect supernatant (chromatin fraction). Cytoplasmic, nuclear, and chromatin fractions were quantitated by BCA.

### Antibodies

The antibodies in **Table 1** were used in the experiments carried out.

**Table 1.** Antibodies used in this paper.

Antibody	Supplier	Western dilution
β-Actin	Sigma Aldrich	1 in 3000
CDK6	Santa Cruz	1 in 200
γH2AX (Ser139)	Cell Signaling	1 in 1000
Lamin a/c	Santa Cruz	1 in 500
MEK2	BD Biosciences	1 in 3000
PP1α	Bethyl Laboratory	1 in 1000
PP1β	Bethyl Laboratory	1 in 1000
PP1γ	Santa Cruz	1 in 200
p53	Oncogene	1 in 500
pATM (Ser1981)	Epitomics	1 in 500
p-p53 (Ser15)	Cell Signaling	1 in 500
Rad21 (SCC1)	abcam	1 in 1000
Rb	Santa Cruz	1 in 200
p73	Santa Cruz	1 in 100
p63	abcam	1 in 1000
p63(TA)	bioLegend	1 in 1000

### Densitometry

Scanned blot was analyzed by Image J (National Institutes of Health). The densitometric values for protein of interest were calculated and normalized to their loading control.

### RNA extraction and reverse transcription

TRIzol Reagent (Life Technologies) was used to extract total RNA following manufacturer's instructions. The extracted RNA was polyadenylated by mixing 1–10ug of RNA diluted up to 20 μl in RNase free water, 6 μl of 5 x First-Strand Buffer (Life Technologies), 3 μl of 10 mM adenosine triphosphate (ATP) (New England Biolabs), and 1 μl of *E. coli* poly (A) polymerase (New England Biolabs) and incubating at 37°C for 60 min followed by 65°C incubation for 20 min. The same amount of RNA was used for all samples within the same experiment. For reverse transcription, 1 μl of Oligo (dT) adapter (0.25 μg/μl), 3 μl of Random Hexamers (0.4 μg/μl) (Promega), 1 μl of 10 mM dNTP mix (Life Technologies), 2 μl of 5 x First-Strand Buffer (Life Technologies), 2 μl of 0.1 M DTT, and 1 μl of Super Script II reverse transcriptase (Life Technologies) were mixed together. The mixture and 30 μl of the polyadenylated RNA solution were mixed together and incubated for 50 min at 42°C, followed by 70°C incubation for 10 min. The following Oligo (dT) adapter primer was used in the reaction:

5′GCGAGCACAGAATTAATACGACTCACTATAGGTTTTTTTTTTTTVN3′

### qRT-PCR

PCR mix: cDNA, 0.5 uM of forward and reverse primers and SYBR Green I master mix (Roche) was plated on LightCycler 480 Multiwell Plate 96 (Roche). All reactions were carried out in triplicates, and the same amount of cDNA was used for all samples in the same experiment. Triplicate wells without cDNA were prepared per plate as a no template control. Internal control was studied per plate, which were GAPDH and U6 snRNA, for mRNA and miRNA respectively. The plates were sealed by a sealing foil (Roche), and were loaded on to LightCycler 480 instrument (Roche). The pre-programmed PCR protocol on LightCycler 480 instrument specific for SYBR Green I Master using a LightCycler 480 Multiwell Plate 96 was used. Comparative Ct method was used to quantify the expression of mRNA and miRNA of interest. The details of primers used are provided in supplementary table.

### Plasmid construct

PP1γ 3′UTR was amplified from genomic DNA (gDNA) extracted from CAL51 using DNAzol (Life Technologies) following manufacture's instructions. Construct was generated by sub-cloning PP1γ 3′UTR into XhoI (5′) and NotI (3′) restriction sites of psiCHECK2 plasmid using T4 Ligase (New England Biolabs). Mutant plasmid was produced by site-directed mutagenesis through running PCR reactions using forward and reverse primer with the desired mutations using the Quik-Change SiteDirect Mutagenesis kit (Stratagene).The insert identity was validated by sequencing. The primers used for mutagenesis were: sense, 5′GCGATCGCTCGAGATGTC*A*T*C*TT*CGAGGCTAG *TAGTCG3′ antisense, 5′CGACTA*CTAGCCTCG*AA*G*A*T*GACATCTCGAGCGATCGC3′ (the mutated nucleotides are underlined and italicized).

### 3′UTR luciferase reporter assay

psiCHECK2-PP1γ 3′UTR WT or mt and miR-34a or negative control miRNA were transfected into CAL51 cells with Lipofectamine 2000™. The transfected cells ready for analysis were replated into 12-well plate at least one day before the assay. Luminescent signal was measured using Dual-Glo Reagent (Promega) following manufacturer's instructions. The luminescence measurement was taken by Luminometer (BMG LABTECH). The data was analyzed by calculating the ratio of luminescence from the experimental Renilla reporter to luminescence from the control Firefly reporter. The ratio was normalized to the ratio of control samples.

### Colony formation assay

Colony formation assay was performed as previously described.^36^ Briefly, cells were plated at an optimal concentration for colonies to form. The plates were incubated until they reach the optimal concentration. To stain the colonies with crystal violet, the plates were first washed PBS. The cells were then fixated with 4% formaldehyde for 20 min at room temperature. Fixative was removed from the wells, washed once with water, and the plates were dried. 0.1% crystal violet (Sigma-Aldrich) was dispensed to the dried wells, incubated for 20 min, then the crystal violet solution was removed and the plates were washed with water for 3 times. The plates were dried and the colonies were counted and analyzed.

### Statistical analysis

All statistical analysis was performed using Graph Pad Prism version 5.00 software (Graph Pad Software, Inc.).
