# Supplementary material for: Micro(mi) RNA-34a targets protein phosphatase (PP)1γ to regulate DNA damage tolerance
Source: Cell Cycle. 2015 Jun 25;14(24):3830–41. doi: 10.1080/15384101.2015.1064202 (PMC4825746; doi:10.1080/15384101.2015.1064202)
Supplement: 1064202_SUPPLEMENTAL_MATERIAL.pdf [file kccy-14-24-1064202-s001.pdf]

SUPPLEMENTAL MATERIAL

Micro(mi) RNA-34a targets protein phosphatase (PP)1γ to regulate DNA damage tolerance

Yuko Takeda & Ashok R. Venkitaraman

Supplemental figure legends.

Supplemental Table 1. Positions of miR-34a seed match sequences in the 3'UTR of PP1γ.

Figure S1. (A) Lysates of cells treated with PP1γ siRNA were used to validate the specificity of the anti- PP1γ antibody by western blot in comparison to Luc siRNA transfected controls. Ponceau staining was used as a loading control. (B) qRT-PCR of PP1γ and CDK6 mRNA expression. cDNA from CAL51 cells 48 h after exposure to 50 nM miR-34a mimic, or transfection with Luc siRNA was used as a substrate for the RT-PCR reactions. The plotted values show the mean ±SEM (one-way ANOVA with Dunett's post-test; ****P*<0.001) (n=2). (C) Representative qRT-PCR of miR-34a in cDNA prepared from CAL51 cells transfected with 50 nM or 75 nM miR-34a mimic. Luc siRNA was transfected as a negative control, as well as to equalize the molarity of miR-34a mimic transfection. Two different timepoints: 48 h and 72 h were studied (one-way ANOVA with Dunett's post-test; ****P*<0.001) (n=1; ±SD)

Figure S2. Damage-induced miR-34a expression is dependent on p53. (A) CAL51 cells were transfected with 30nM p53 deconvoluted siRNA (Qiagen), before extract preparation and Western blotting for p53 expression at the indicated times. (B) Experimental scheme: CAL51 cells were plated 24 h prior to siRNA transfection. The cells were transfected with 30nM p53 deconvoluted siRNA (Qiagen), irradiated at 3 Gy and harvested 72 h post irradiation. (C) qPCR analysis of miR-34a expression after exposure to 3 Gy IR, following treatment with control or p53 siRNA. p<0.01(**) by one-way ANOVA with Bonferroni's post-test. n=3, ± SEM. (D) p53 activation by Ser15 phosphorylation was determined 4h and 72h after IR in cells treated with control or p53 siRNA in the same experiment shown in panel (C).

Supplemental materials and methods.

Details of siRNA and miRNA mimic/inhibitor:

siRNA/miRNA	Detail	Manufacturer
negative control siRNA	Luciferase siRNA	Eurofins MWG Operon
negative control miRNA	cel-miR-67	Dharmacon
miR-34a mimic	hsa-miR-34a mimic	Dharmacon
miR-34a inhibitor	hsa-miR-34a inhibitor	Dharmacon
SMARTpool: ON-TARGET plus PP1γ	PP1γ pooled siRNA	Dharmacon
Set of 4: ON-TARGETplus TP63 siRNA	p63 deconvoluted siRNA	Dharmacon
Set of 4: ON-TARGETplus TP73 siRNA	p73 deconvoluted siRNA	Dharmacon
Hs_TP53_7 FlexiTube	p53 deconvoluted siRNA	Qiagen
Hs_TP53_9 FlexiTube	p53 deconvoluted siRNA	Qiagen
FlexiTube GeneSolution GS8626 for TP63	p63 deconvoluted siRNA	Qiagen
FlexiTube GeneSolution GS7161 for TP73	p73 deconvoluted siRNA	Qiagen

Details of primers used for qRT-PCR experiments:

Gene	Primer direction	Sequence (5'->3')
CDK6	F	TGCACAGTGTCACGAACAGA
	R	ACCTCGGAGAAGCTGAAACA
PPP1CC (PP1γ)	F	TGGCGTGGGAAAGGAGGTGTGA
	R	GACCCTCTCACTTCCAGCAGCCGT
U6 snRNA	F	CGCTTCGGCAGCACATATAC
	R	TTCACGAATTTGCGTGTCAT
GAPDH	F	AGCCACATCGCTCAGACAC
	R	GCCCAATACGACCAAATCC
miR-34a	F	TGGCAGTGTCTTAGCTGGTTGT
Universal reverse	R	GCGAGCACAGAATTAATACGACTCAC

<table>
<tr><th>Gene</th><th>Position of miRNA seed match sequence in 3’UTR</th><th colspan="2">miR-34a and target seed match</th></tr>
<tr><td>PPP1CC</td><td>13-19</td><td>hsa-miR-34a</td><td>3’ UGUUGGUCGAUUCUGUGACGGU 5’</td></tr>
<tr><td></td><td></td><td></td><td>| | | | | | | | |</td></tr>
<tr><td></td><td></td><td>PPP1CC</td><td>5’...NNAUGUCGUUUUGACACUGCCU... 3’</td></tr>
<tr><td>ANK3</td><td>34-40</td><td>hsa-miR-34a</td><td>3’ UGUUGGUCGAUUCUGUGACGGU 5’</td></tr>
<tr><td></td><td></td><td></td><td>| | | | | | |</td></tr>
<tr><td></td><td></td><td>ANK3</td><td>5’...GGAUCAUAAGUUUUUACUGCCA... 3’</td></tr>
<tr><td></td><td>545-551</td><td>hsa-miR-34a</td><td>3’ UGUUGGUCGAUUCUGUGACGGU 5’</td></tr>
<tr><td></td><td></td><td></td><td>| | | | | | |</td></tr>
<tr><td></td><td></td><td>ANK3</td><td>5’...AAAUGUUGAUAAGGCACUGCCU... 3’</td></tr>
<tr><td></td><td>1984-1991</td><td>hsa-miR-34a</td><td>3’ UGUUGGUCGAUUCUGUGACGGU 5’</td></tr>
<tr><td></td><td></td><td></td><td>| | | | | | | |</td></tr>
<tr><td></td><td></td><td>ANK3</td><td>5’...GGGUCUACAGGUAUCACUGCCA... 3’</td></tr>
<tr><td>MYRIP</td><td>14-21</td><td>hsa-miR-34a</td><td>3’ UGUUGGUCGAUUCUGUGACGGU 5’</td></tr>
<tr><td></td><td></td><td></td><td>| | | | | | | |</td></tr>
<tr><td></td><td></td><td>MYRIP</td><td>5’...NCACCAUGGAAUUCCACUGCCA... 3’</td></tr>
<tr><td></td><td>28-34</td><td>hsa-miR-34a</td><td>3’ UGUUGGUCGAUUCUGUGACGGU 5’</td></tr>
<tr><td></td><td></td><td></td><td>| | | | | | |</td></tr>
<tr><td></td><td></td><td>MYRIP</td><td>5’...ACUGCCAGUGACC-CACUGCCU... 3’</td></tr>
<tr><td>PKP4</td><td>694-701</td><td>hsa-miR-34a</td><td>3’ UGUUGGUCGAUUCUGUGACGGU 5’</td></tr>
<tr><td></td><td></td><td></td><td>| | | | | | |</td></tr>
<tr><td></td><td></td><td>PKP4</td><td>5’...UUCAUAGGACACUUCACUGCCA... 3’</td></tr>
<tr><td>RALGPS2</td><td>203-209</td><td>hsa-miR-34a</td><td>3’ UGUUGGUCGAUUCUGUGACGGU 5’</td></tr>
<tr><td></td><td></td><td></td><td>| | | | | | |</td></tr>
<tr><td></td><td></td><td>RALGPS2</td><td>5’...GGAGUUGCAAAACAAACUGCCA... 3’</td></tr>
<tr><td></td><td>249-255</td><td>hsa-miR-34a</td><td>3’ UGUUGGUCGAUUCUGUGACGGU 5’</td></tr>
<tr><td></td><td></td><td></td><td>| | | | | | |</td></tr>
<tr><td></td><td></td><td>RALGPS2</td><td>5’...UGACCUGUGGAAACCACUGCCU... 3’</td></tr>
<tr><td>STRN3</td><td>57-64</td><td>hsa-miR-34a</td><td>3’ UGUUGGUCGAUUCUGUGACGGU 5’</td></tr>
<tr><td></td><td></td><td></td><td>| | | | | | | |</td></tr>
<tr><td></td><td></td><td>STRN3</td><td>5’...AAGAGGGUCUGCAUCACUGCCA... 3’</td></tr>
<tr><td>ARID4B</td><td>272-278</td><td>hsa-miR-34a</td><td>3’ UGUUGGUCGAUUCUGUGACGGU 5’</td></tr>
<tr><td></td><td></td><td></td><td>| | | | | | | |</td></tr>
<tr><td></td><td></td><td>ARID4B</td><td>5’...AAUGGAGGCUUUU-CACUGCCU... 3’</td></tr>
<tr><td>JMJD1C</td><td>482-488</td><td>hsa-miR-34a</td><td>3’ UGUUGGUCGAUUCUGUGACGGU 5’</td></tr>
<tr><td></td><td></td><td></td><td>| | | | | | |</td></tr>
<tr><td></td><td></td><td>JMJD1C</td><td>5’...CAUAAUCAGAAAAUUACUGCCA... 3’</td></tr>
</table>

Supplemental Table 1

A

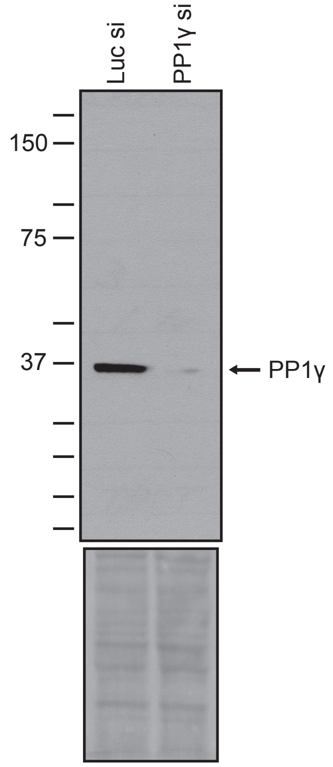

B

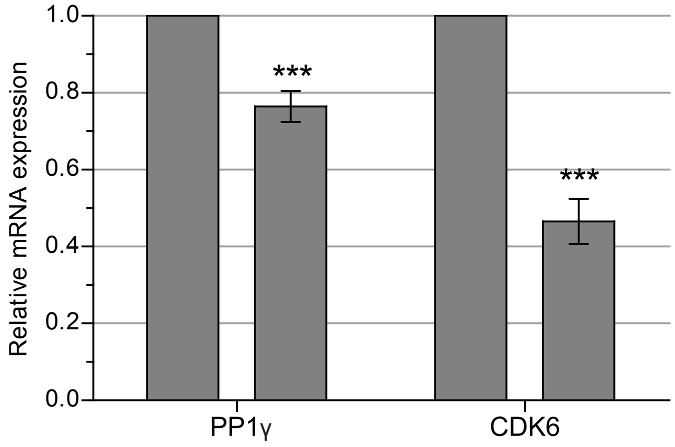

C

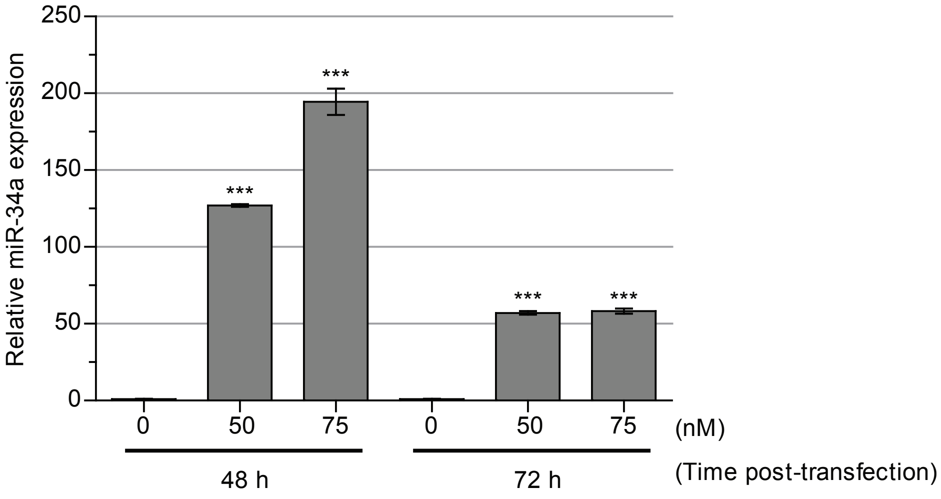

Supplemental Figure 1

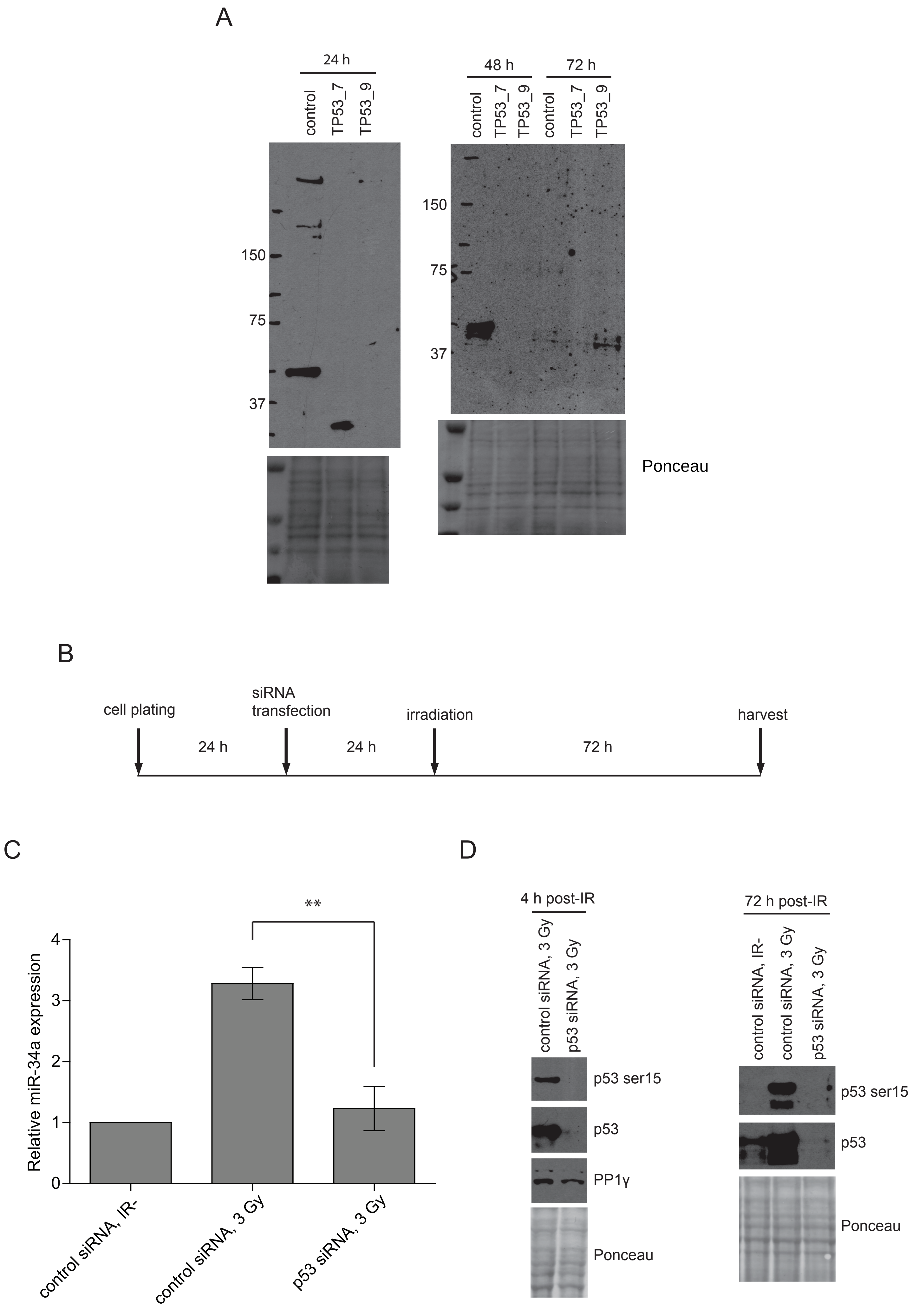

Supplemental Figure 2
